# Aldo Keto Reductase 1B7 and Prostaglandin F_2α_ Are Regulators of Adrenal Endocrine Functions

**DOI:** 10.1371/journal.pone.0007309

**Published:** 2009-10-07

**Authors:** Sarah Lambert-Langlais, Jean-Christophe Pointud, Anne-Marie Lefrançois-Martinez, Fanny Volat, Michèle Manin, François Coudoré, Pierre Val, Isabelle Sahut-Barnola, Bruno Ragazzon, Estelle Louiset, Catherine Delarue, Hervé Lefebvre, Yoshihiro Urade, Antoine Martinez

**Affiliations:** 1 CNRS, UMR6247 - Genetic, Reproduction & Development (GReD), Clermont University, Aubière, France; 2 Laboratoire Pharmaco-Toxicologie, Centre de Biologie, CHU G. Montpied, Clermont-Ferrand, France; 3 INSERM U567,CNRS UMR8104, Department of Endocrinology Metabolism & Cancer, Cochin Institut, Paris, France; 4 INSERM U413, Laboratory of Cellular and Molecular Neuroendocrinology, Rouen University, Mont-Saint-Aignan, France; 5 Department of Molecular Behavorial Biology, Osaka Bioscience Institute, Osaka, Japan; Mayo Clinic College of Medicine, United States of America

## Abstract

Prostaglandin F_2α_ (PGF_2α_), represses ovarian steroidogenesis and initiates parturition in mammals but its impact on adrenal gland is unknown. Prostaglandins biosynthesis depends on the sequential action of upstream cyclooxygenases (COX) and terminal synthases but no PGF_2α_ synthases (PGFS) were functionally identified in mammalian cells. *In vitro*, the most efficient mammalian PGFS belong to aldo-keto reductase 1B (AKR1B) family. The adrenal gland is a major site of AKR1B expression in both human (AKR1B1) and mouse (AKR1B3, AKR1B7). Thus, we examined the PGF_2α_ biosynthetic pathway and its functional impact on both cortical and medullary zones. Both compartments produced PGF_2α_ but expressed different biosynthetic isozymes. In chromaffin cells, PGF_2α_ secretion appeared constitutive and correlated to continuous expression of COX1 and AKR1B3. In steroidogenic cells, PGF_2α_ secretion was stimulated by adrenocorticotropic hormone (ACTH) and correlated to ACTH-responsiveness of both COX2 and AKR1B7/B1. The pivotal role of AKR1B7 in ACTH-induced PGF_2α_ release and functional coupling with COX2 was demonstrated using over- and down-expression in cell lines. PGF_2α_ receptor was only detected in chromaffin cells, making *medulla* the primary target of PGF_2α_ action. By comparing PGF_2α_-responsiveness of isolated cells and whole adrenal cultures, we demonstrated that PGF_2α_ repressed glucocorticoid secretion by an indirect mechanism involving a decrease in catecholamine release which in turn decreased adrenal steroidogenesis. PGF_2α_ may be regarded as a negative autocrine/paracrine regulator within a novel intra-adrenal feedback loop. The coordinated cell-specific regulation of COX2 and AKR1B7 ensures the generation of this stress-induced corticostatic signal.

## Introduction

The aldose reductases (AKR1Bs) belong to the aldo-keto reductase (AKR) superfamily that contains more than 114 proteins expressed in prokaryotes and eukaryotes. This superfamily performs oxidoreduction of a wide variety of endogenous and exogenous substrates including aldoses, aliphatic and aromatic aldehydes and ketones, monosacharides and prostaglandins [1]. Among the 12 members of the AKR1B subfamily, aldose reductase (AKR1B1 in human and AKR1B3 in mouse) has been a focus of interest because of its role in the development of secondary diabetic complications but its physiological functions remain poorly understood [2].

Aldose reductase deficient mice (*Akr1b3* ^−/−^) exhibit a partially defective urine-concentrating ability [3], [4] and renal structural abnormalities [5]. AKR1B1/B3 is also present in a broad range of tissues and other physiological functions based on its enzymatic activities *in vitro* have been proposed. Notably, AKR1B1/B3 is thought to be involved in detoxification of harmful cellular metabolites including 4-hydroxynonenal [6] and isocaproaldehyde [7]. But this function remains to be demonstrated *in vivo* since AKR1B3 deficient mice appeared to be healthy. It is likely that the detoxification function may be taken over by other AKR1B subfamily members. Indeed, we and others have identified AKR1B7 [8], [9] and AKR1B8 [10] as two other murine members of the family and AKR1B10 has been isolated in human small intestine [11], [12].

AKR1B7 presents two interesting characteristics: a tissue-restricted expression (*vas deferens*, steroidogenic tissues, small intestine and adipose tissue) and a tissue-specific hormonal regulation [13]–[19]. In the murine adrenal cortex, the *Akr1b7* gene is up-regulated at the transcriptional level by ACTH [20], [21], [22] and acts as a major reductase for isocaproaldehyde formed during steroidogenesis [23]. In human adrenocortical cells, AKR1B1 was proposed to undertake the same function as AKR1B7 in murine species, based on its hormonal sensitivity and isocaproaldehyde reductase activity [24]. Interestingly, Madore and colleagues [25] proposed that the bovine 20α-hydroxysteroid dehydrogenase also known as AKR1B5 had a prostaglandin F_2α_ synthase (PGFS) activity in the endometrium. Furthermore, we have established by *in vitro* studies, that this property can be extended to other but not all AKR1B enzymes since AKR1B1, AKR1B3 and AKR1B7 were shown to catalyze the reduction of prostaglandin H_2_ (PGH_2_) into PGF_2α_ whereas AKR1B8 and AKR1B10 recombinant proteins were devoid of such activity [26]. Importantly, based on their kinetic parameters, recombinant AKR1B1, AKR1B3 and AKR1B7 display better PGFS activities than the putative PGFS that were previously characterized in mammals.

Prostaglandins are paracrine/autocrine cell mediators sharing a common precursor, PGH_2_, which is synthesized from free arachidonic acid by the cyclooxygenases type 1 (COX1) or type 2 (COX2). COX1 is regarded as a constitutively expressed enzyme. COX2 on the other hand is undetectable in most tissues in basal conditions but can be induced by various mitogenic agents and inflammatory stimuli (for review see [27]). These different regulations imply that COX1- and COX2-dependent PG productions will be primarily required for housekeeping functions and rapid adaptation to various stimuli, respectively. However, the identity and regulation of downstream enzymes involved in the selective production of each isotype of prostaglandins remain largely unknown. PGF_2α_ is the main initiator of labour and regression of corpus luteum (luteolysis) in most mammals (for review see [28]). Two of the hallmarks features of luteolysis are the decrease in progesterone synthesis and programmed luteal cell death. PGF_2α_-induced inhibition of progesterone biosynthesis is mediated by the PGF_2α_ receptor (FP receptor) present in luteal cells. Although it has a primary role in reproductive function, the cellular and molecular mechanisms by which PGF_2α_ is produced in living cells and its role in other steroidogenic tissues remain largely unknown [29], [30]. AKR1Bs with PGFS activity are highly expressed and for some of them specifically regulated in the adrenal cortex. We thus decided to explore the capacity of the adrenal to produce PGF_2α_ and to investigate the effect that this production has on the endocrine functions of the gland.

## Results

### Hormonal regulation and differential expression of AKR1Bs and COX enzymes in adrenal tissue

**Figure 1:**
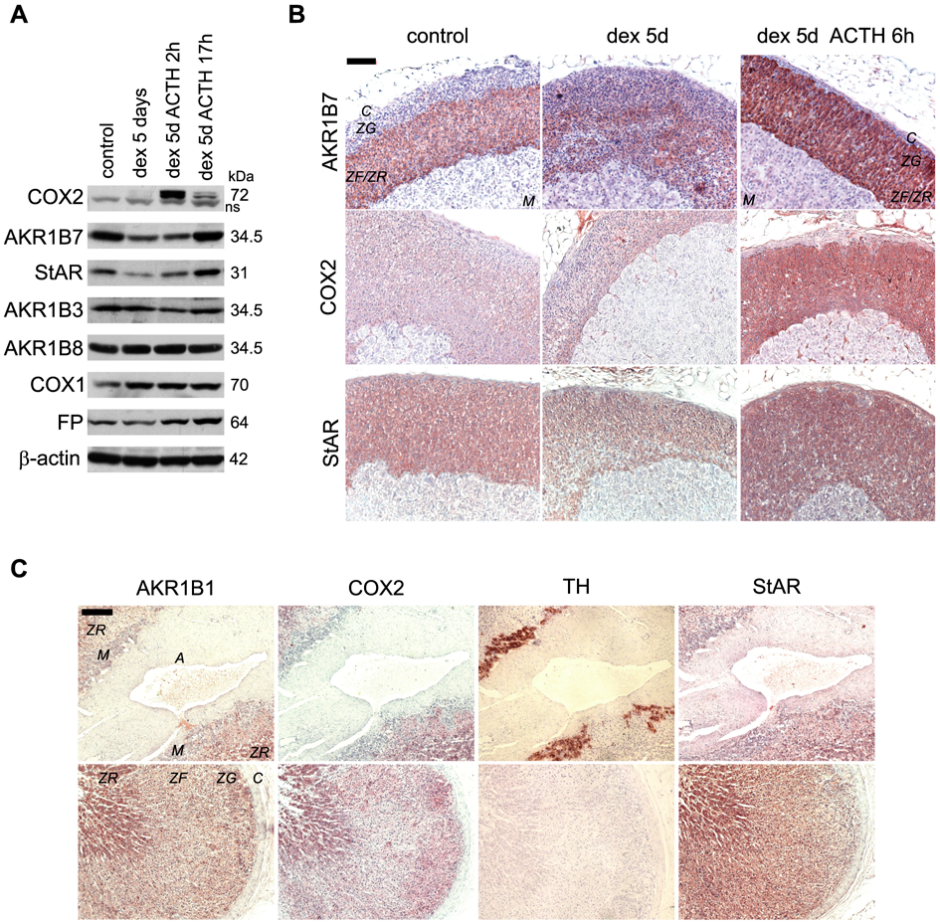
Hormonal regulation and differential expression of AKR1Bs, COXs and FP receptor proteins in adrenal glands. *A*, Differential expression of AKR1Bs, COXs and FP receptor proteins in response to ACTH in mouse adrenal glands. Protein extracts (30 µg/lane) from pooled adrenal glands (3 to 6 animals per condition) treated with vehicle alone (5 days, control), dexamethasone (5 days, dex) or dexamethasone (5 days) plus ACTH for time ranging from 2 to 17 h (dex ACTH) were subjected to western blot analysis. Molecular weight markers are shown on the right. COX2 multiple molecular species are a consequence of the heterogeneous glycosylation of the protein (ns, non specific signal). *B*, Immunolocalization of AKR1B7, COX2 and StAR in mouse adrenal sections. Sections of adrenal glands from male mice treated 5 days with vehicle (control), 5 days with dexamethasone (dex) or with dexamethasone plus ACTH for the last 6 h (dex + ACTH) were immunostained with anti-AKR1B7 (L3 antiserum), anti-COX2 and anti-StAR (steroidogenic cell marker) antibodies (*B*, *bar*, 100 µm). *C*, immunolocalization of AKR1B1, COX2, TH and StAR in human adrenal sections. Sections of normal human adrenal glands were incubated with anti-AKR1B1 (L3 antiserum), anti-COX2, anti-StAR (steroidogenic cell marker) and anti-tyrosine hydroxylase (TH) (chromaffin cell marker) antibodies (*C*, *bar*, 200 µm). *A, artery, M, medulla, ZG, zona glomerulosa, ZF, zona fasciculata, ZR, zona reticularis, C, capsule*.

We first investigated the effect of modulations of the hypothalamo-pituitary-adrenal (HPA) axis on the capacity of mouse adrenal glands to synthesise and respond to PGF_2α_. For this, whole adrenal protein extracts were submitted to western blot analysis (Fig. 1A). As expected, the level of StAR protein (an ACTH-regulated marker of steroidogenic activity) was decreased under dexamethasone-mediated HPA axis blockade (low plasma ACTH) and progressively restored 17 h after the injection of exogenous ACTH. As previously described, the levels of the ACTH-responsive aldose reductase-like AKR1B7 followed the same pattern, confirming the efficiency of the hormonal treatments [31]. By contrast, AKR1B3 and AKR1B8 proteins were unresponsive to any of the treatments and exhibited constitutive expressions. Interestingly, the rate-limiting enzymes of the prostaglandins synthesis pathway, COX1 and COX2, were both expressed in the adrenals but showed very different hormonal sensitivity. COX1 was constitutively expressed. By contrast, COX2 was undetectable in basal condition. It was strongly stimulated, 2 h after ACTH injection. This stimulation was transient, as shown by the reduction of COX2 accumulation 17 h after ACTH administration. In contrast, the PGF_2α_ receptor FP was constitutively expressed in the adrenal. These data suggest that mouse adrenal glands have the capacity to synthesize PGF_2α_ in either a constitutive or an ACTH-dependent manner. The presence of FP indicates that they might be able to respond to this signal directly.

Expression of AKR1B7, AKR1B1 and COX2 in mouse and human adrenal glands was then analysed by immunohistochemistry (Fig. 1B). AKR1B7 was detected at high levels in the *zona fasciculata* of the cortex in basal conditions. This level was decreased upon dexamethasone treatment. AKR1B7 staining increased dramatically and expanded throughout the whole cortex in the presence of ACTH (Fig. 1B). StAR protein had a similar expression pattern, although it spanned the entire cortex irrespective of the hormonal status. In contrast, COX2 positive cells were only found in the adrenal cortex of ACTH-stimulated mice. In human adrenal sections, AKR1B1 and COX2 were restricted to the steroidogenic cells of the cortex (StAR-positive cells) and were absent from the chromaffin cells of the *medulla* (tyrosine hydroxylase positive cells) (Fig. 1C).

**Figure 2:**
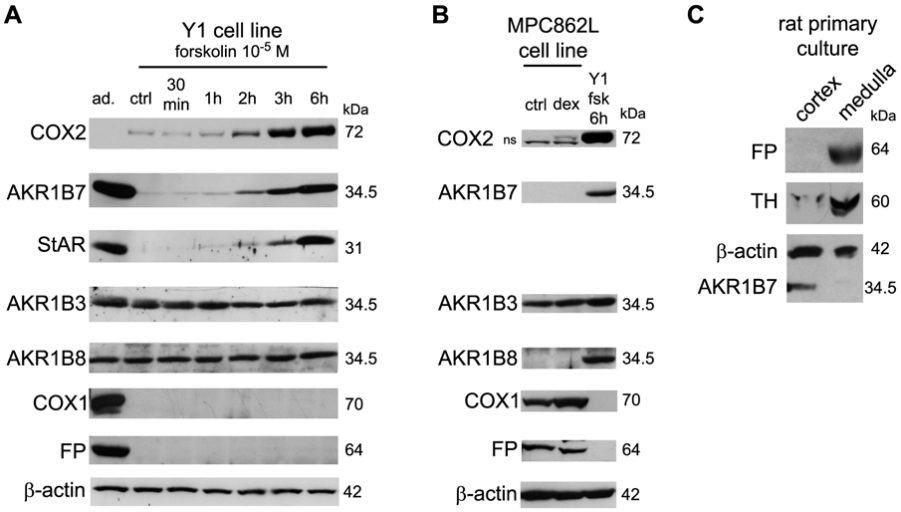
Differential expression of AKR1Bs, COXs and FP receptor proteins in cell cultures from adrenal cortex and from adrenal *medulla*. *A*, Time-dependent response to 10^−5^ M forskolin treatment of StAR, AKR1Bs, COXs and FP receptor proteins levels in adrenocortical Y1 cell line was analyzed by western blot. Whole adrenal protein extract was used as a positive control, (ad.). *B*, Expression levels of AKR1Bs, COXs and FP receptor proteins were analyzed by western-blot in the chromaffin MPC862L cell line either untreated (ctrl) or treated with 10^−6^ M dexamethasone (dex) for 12 h. These levels were compared to the levels observed in Y1 cells stimulated by 10^−5^ M forskolin (Fsk) for 6 h (ns, non specific signal). *C*, FP receptor expression in primary cell cultures of rat adrenal cortical and medullary cells. AKR1B7 and TH positive signals were used as markers of steroidogenic and chromaffin identity, respectively. Molecular weights are indicated on the right.

In order to define which of the adrenal cell types was responsible for PGF_2α_ production and which cell type could respond to this signal, we performed western blot experiments with both steroidogenic cortical and medullary chromaffin cell lines (Fig. 2). In contrast with whole adrenal protein extracts (ad.), both FP receptor and COX1 were absent from the murine Y1 adrenocortical cell line (Fig. 2A). In these cells, AKR1B3 and AKR1B8 were constitutively expressed. Remarkably, COX2 and AKR1B7 levels showed parallel time-dependent increases upon forskolin treatment, an activator of cAMP synthesis that stimulates steroidogenesis and StAR expression. The murine MPC862L chromaffin cell line was cultured in the absence or in the presence of dexamethasone in order to mimick the known stimulatory action of glucocorticoids on catecholamine production. In contrast with cortical cells, chromaffin MPC862L cells expressed constitutive high levels of FP receptor and COX1 whereas COX2 was either absent or expressed at very low levels (Fig. 2B). The aldose reductase AKR1B3 was constitutively expressed in these cells whereas AKR1B7 and AKR1B8 were undetectable. We confirmed the mutually exclusive expression of AKR1B7 (cortical cells) and FP receptor (chromaffin cells) by western blots analyses performed on primary cell cultures from dispersed rat adrenals (Fig. 2C).

Altogether our data demonstrated that there was a coordinated cell-specific expression of COX2 and AKR1B7/B1 in the steroidogenic cells of the adrenocortical cortex. This suggested that this part of the gland had the potential to produce PGF_2α_ under cAMP/ACTH control.

### Functional coupling between COX2 and AKR1B7

**Figure 3:**
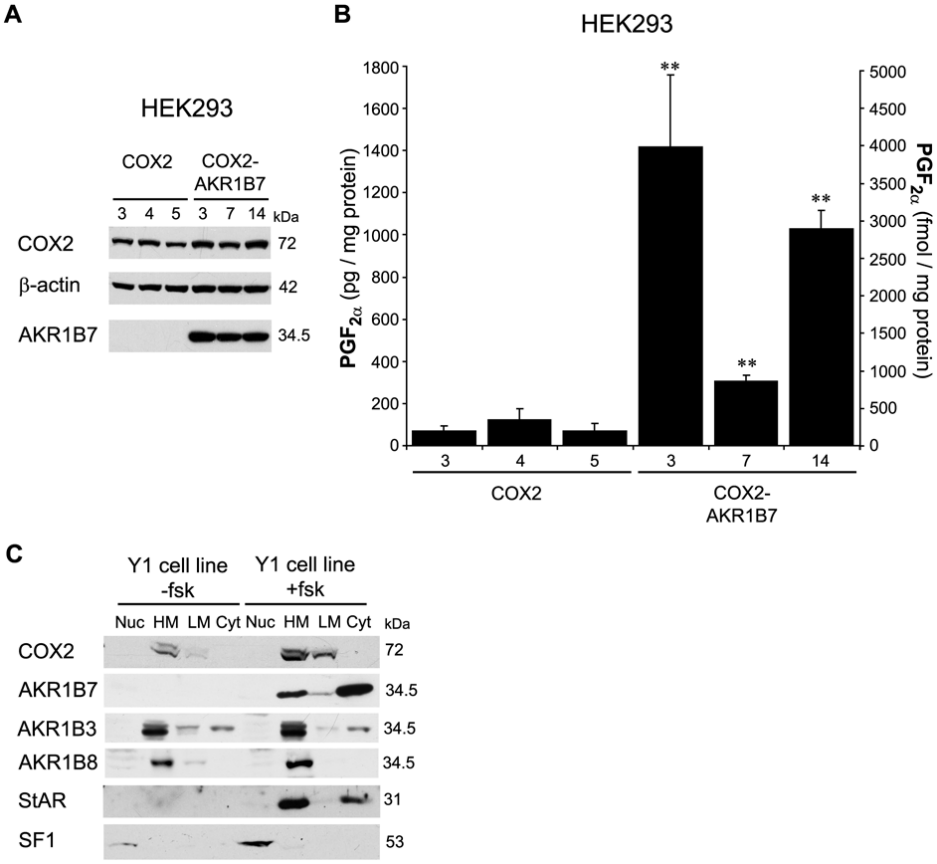
Functional cellular coupling between COX2 and AKR1B7. *A*, Western blot analysis of six different HEK293 clones stably transfected with COX2 expression vector in combination with empty vector (COX2, clones 3, 4, 5) or AKR1B7 expression vector (COX2-AKR1B7, clones 3, 7, 14). *B*, ELISA quantification of PGF_2α_ in media from stably transfected HEK293 cell clones, expressing COX2 alone (COX2-3, -4, -5) or in combination with AKR1B7 (COX2-AKR1B7-3, -7, -14). Cells were stimulated for 30 min with 10 µM A23187 ionophore and culture media were used for PGF_2α_ quantification. Values were expressed as the mean of 4 experiments ± S.D. *Asterisks* point values significantly different from the release of PGF_2α_ by the COX2-4 cell clone. *, *P*<0.05, **, *P*<0.01. *C*, Subcellular localization of AKR1B7, AKR1B3, AKR1B8 and COX2 in Y1 adrenocortical cells was analysed by western-blot. Protein extracts (40 µg/lane) from nuclear (Nuc), heavy membrane (HM), light membrane (LM) and cytosolic fractions (Cyt) of Y1 adrenocortical cells untreated or treated with 10^−5^ M forskolin for 6 h were subjected to western blot analysis. StAR and SF1 signals were used as markers of heavy membrane fraction and nuclear fraction respectively. Molecular weight markers are shown on the right.

In order to assess a functional coupling between COX2 and AKR1B7, we stably transfected plasmids encoding these two enzymes into the HEK293 cell line that is known to be devoid of COX activity [32] (Fig. 3A, B). Expression of COX2 and AKR1B7 was confirmed by western blotting (Fig. 3A). Amounts of PGF_2α_ released in the media by HEK293 clones expressing COX2 alone or both COX2 and AKR1B7 were measured by ELISA (Fig. 3B). A low basal production of PGF_2α_ was detected in similar amounts in three independent COX2-expressing clones while a 5- to 20-fold increase of PGF_2α_ levels was observed in three independent clones upon stable introduction of AKR1B7 expression vector. Functional coupling for prostaglandin synthesis is dependent on a membrane association of both the COX and prostaglandin synthase enzymes [33], [34]. We thus analysed subcellular localization of AKR1Bs and COX by cellular fractionation of Y1 adrenocortical cells in the absence or presence of forskolin (Fig. 3C). As expected, COX2 was only associated with heavy and low membranes fractions in both hormonal conditions and was absent from nuclear extracts or soluble cytosolic fractions. Importantly, the PGF synthases AKR1B7 and AKR1B3 which were previously considered as cytosolic enzymes, colocalized with COX2 in the heavy membranes fraction and to a lesser extent in the light membranes fractions. Whereas AKR1B3 was constitutively present in these two fractions, AKR1B7 was only observed in the presence of forskolin (Fig. 3C). Note that AKR1B8 which was shown to be devoid of PGF synthase activity [26] was found associated with heavy membranes in all conditions. StAR, a mitochondrial protein responsive to cAMP was mainly found associated with the heavy membrane fraction upon forskolin stimulation. On the other hand, the steroidogenic transcription factor SF-1, was found in the nuclear fraction. This validated the subcellular fractionation procedure. Altogether, we concluded that there was a functional cellular coupling between COX2 and AKR1B7 that resulted in PGF_2α_ synthesis.

### Hormonal sensitivity of PGF_2α_ production in adrenocortical cells and the role of AKR1B7

**Figure 4:**
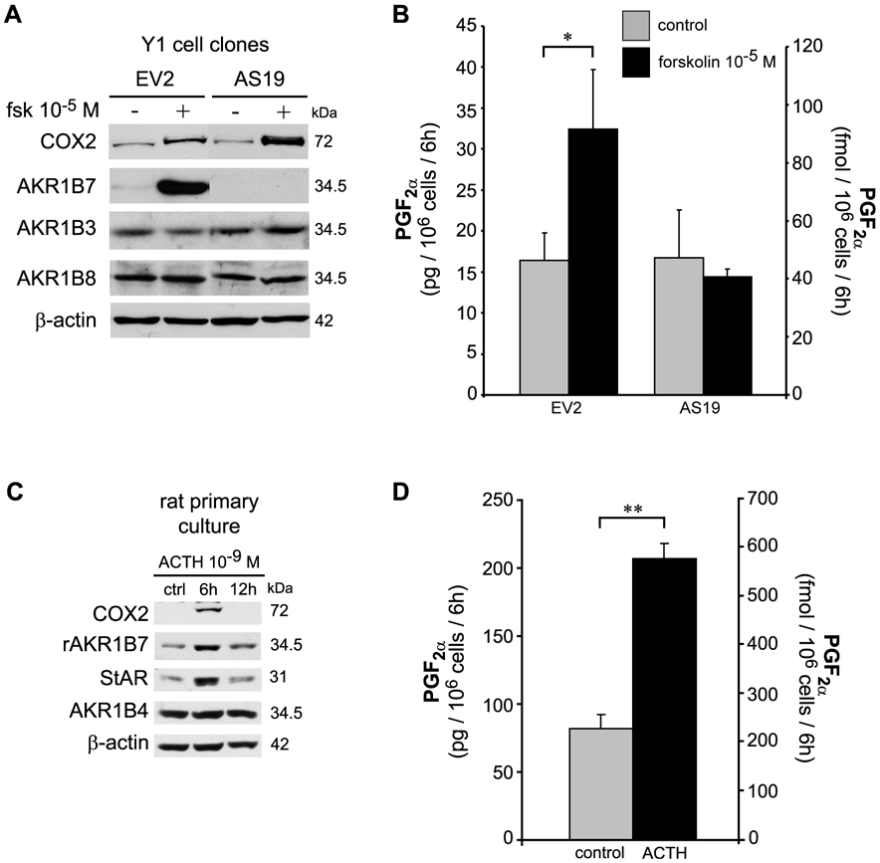
Hormonal sensitivity of PGF_2α_ production in rodent adrenocortical cells and involvement of AKR1B7 protein in PGF_2α_ production. *A*, Western-blot analysis of AKR1Bs and COX2 proteins accumulation in stably transfected Y1 cell clones expressing AKR1B7 (empty vector EV2) or devoid of AKR1B7 (antisense vector AS19) untreated or treated with 10^−5^ M forskolin (Fsk) for 6 h. Molecular weight markers are shown on the right. *B*, ELISA quantification of PGF_2α_ in media from stably transfected Y1 cell clones expressing AKR1B7 (EV2) or devoid of AKR1B7 (AS19), untreated or treated with 10^−5^ M forskolin for 6 h. *C*, Differential expression of AKR1Bs and COX2 proteins in primary cultures of rat cortical cells treated with vehicle (ctrl) or with 10^−9^ M ACTH for 6 h or 12 h. *D*, ELISA quantification of PGF_2α_ release in media from rat adrenocortical primary cells, cultured in the absence or presence of 10^−9^ M ACTH for 6 h. Values are the mean of 3 experiments ± S.D. *, *P*<0.05, ** *P*<0.01.

We showed that in Y1 adrenocortical cells, COX2 and AKR1B7 were associated in the same membrane fractions under hormonal stimulation. We thus asked whether cAMP would stimulate PGF_2α_ production in a manner dependent on the presence of AKR1B7. In order to answer this question we used Y1 cells clones stably transfected with an antisense cDNA targeting AKR1B7 (AS19) or transfected with an empty vector (EV2) [23]. As expected, AKR1B7 was undetectable in the Y1 AS19 clone (antisense). The control EV2 and the antisense AS19 clones had similar levels of expression of AKR1B3, AKR1B8 and COX2 either in basal condition or under forskolin stimulation (Fig. 4A). Both Y1 clones produced similar amounts of PGF_2α_ in basal conditions (Fig. 4B). Forskolin treatment induced a two-fold increase in PGF_2α_ release by Y1 EV2 cells whereas PGF_2α_ production remained at the basal level in the absence of AKR1B7 (Y1 AS19). To confirm that cAMP/ACTH induced an increase in PGF_2α_ release in a non-immortalized cell culture model, we analyzed PGF_2α_ production in primary cultures of rat adrenocortical cells. As shown in Fig. 4C, rat adrenocortical cells showed a transient stimulation of COX2 expression upon ACTH treatment for 6h. ACTH sensitivity of rAKR1B7 and StAR protein accumulation was similar to that observed in Y1 cells, whereas AKR1B4 (the rat ortholog of mouse AKR1B3) was constitutively expressed. Rat adrenocortical cells produced PGF_2α_ in basal condition and showed a 2.5-fold induction of PGF_2α_ release upon ACTH stimulation (Fig. 4D). These experiments demonstrated that the AKR1B7 enzyme was responsible for the cAMP/ACTH-stimulated production of PGF_2α_ by steroidogenic adrenocortical cells.

### Role of PGF_2α_ in adrenal endocrine functions

**Figure 5:**
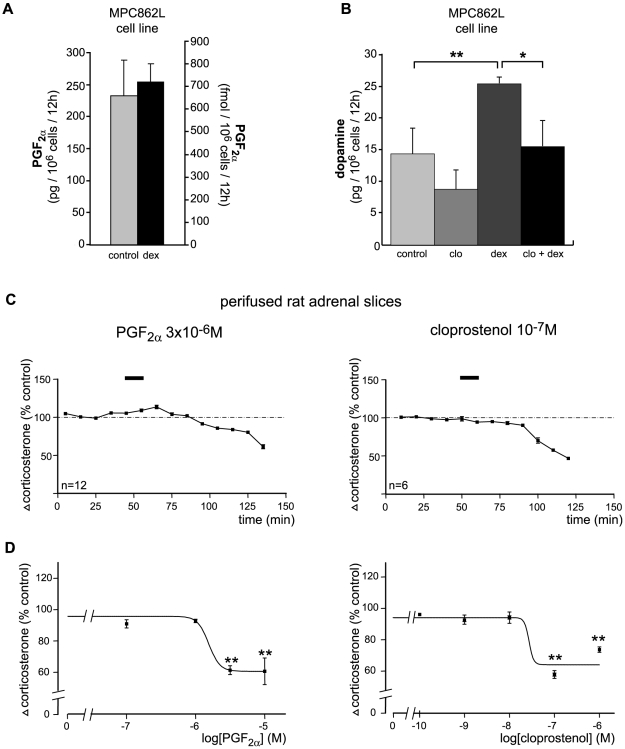
Role of PGF_2α_ in adrenal endocrine functions. *A*, ELISA quantification of PGF_2α_ release by chromaffin MPC862L cells untreated (control) or treated with 10^−6^ M dexamethasone for 12 h (dex). *B*, HPLC quantification of dopamine secretion by MPC862L cells cultured in absence or presence of 10^−7^ M cloprostenol (clo) (PGF_2α_ analogue) used either alone or in combination with 10^−6^ M dexamethasone for 12 h (clo+dex). Values are the mean of 3 experiments ± S.D. *, *P*<0.05, ** *P*<0.01. *C*, Typical perifusion profiles illustrating the effects of increasing concentrations of PGF_2α_ (0.1 µM to 10 µM) and cloprostenol (0.1 nM to 1 µM) on corticosterone secretion. Horizontal bars indicate the start point and duration of PGF_2α_ or cloprostenol infusions. *D*, Semi-logarithmic plot showing the effect of increasing concentrations of PGF_2α_ and cloprostenol on the inhibition of corticosterone secretion. Results are expressed as a percentage of the basal secretory rate. Experimental values were calculated from data similar to those presented in Fig. 5 *C*. Each curve represents the mean ± SEM of 6 to 12 independent experiments. After stabilization, the mean secretion rate of corticosterone in basal condition was 251±14 pg/min per adrenal. The concentration-response curve was fitted using the Prism program (GraphPad Software, Inc., San Diego, CA). **P<0.001.

The experiments illustrated in Fig. 2 provided evidence that the FP receptor was absent from steroidogenic adrenocortical cells (either immortalized cells or cells directly dispersed from adrenal cortex). It is thus likely that PGF_2α_ secreted by these cells might act as a paracrine factor. We have shown that chromaffin cells constitutively expressed high levels of FP receptor, COX1 and AKR1B3. Consistent with this, the MPC862L chromaffin cell line secreted PGF_2α_ into the culture media. This production was insensitive to glucocorticoids (dexamethasone) (Fig. 5A). Glucocorticoids are known stimulators of catecholamine biosynthesis. In order to investigate a possible role of PGF_2α_ on glucocorticoid-induced catecholamine release, we looked at the effects of a PGF_2α_ analogue (cloprostenol) on the dopamine secretion of MPC862L cells in the absence or presence of dexamethasone (Fig. 5B). As expected, dexamethasone induced a 2-fold increase in dopamine release. Interestingly, treatment with cloprostenol (10^−7^ M) not only decreased basal dopamine release but also completely antagonized dexamethasone-induced dopamine secretion. The same effect was obtained on dopamine secretion with 2.10^−6^ M PGF_2α_ (data not shown). These data strongly suggested that PGF_2α_ was exerting an inhibitory effect on chromaffin cells dopamine release, through an autocrine/paracrine mechanism. The concentration and content of PGF_2α_ in mouse adrenal tissue were determined by GC-MS (130±38 pmol/g tissue and 456±130 fmol per gland, respectively) and were in a similar range to levels measured in cell culture systems (Fig. 4–5). When expressed relative to adrenal volume [35], the physiological tissue concentration of PGF_2α_ appeared to be in the µM range.

Cell-cell interactions play a crucial role for maintenance of the homeostasis of adrenal glands (reviewed in [36]). We showed that FP receptor was not expressed in the steroidogenic cells of the adrenal cortex (Fig. 2) and indeed PGF_2α_ had no effect on steroidogenic activity of Y1 cells or rat adrenocortical cells in primary cultures (data not shown). This suggested that PGF_2α_ could not directly influence cortical cell activity. We thus investigated the effect of PGF_2α_ on corticosterone release by setting-up perifusions of isolated rat adrenals, an experimental approach which preserves the architecture of the gland. Various concentrations of PGF_2α_ and of its synthetic analogue, cloprostenol were applied during 20 min. Aliquots of the perifused media were collected every 10 min for determination of corticosterone concentrations (Fig. 5C). PGF_2α_ and cloprostenol induced a dose-dependent inhibition of corticosterone release (Fig. 5D). The half-maximum effective doses (IC_50_) of PGF_2α_ and cloprostenol were 1.58±0.3×10^−6^ M and 2.51±0.2×10^−8^ M, respectively. These doses were in agreement with physiological contents found in the adrenal tissue. Maximum responses for PGF_2α_ (−39.4±6.6%) and cloprostenol (−36±9.3%) were obtained with concentrations of 3×10^−6^ M and 10^−7^ M, respectively (*P*<0.001). The negative effect of PGF_2α_ was restricted to glucocorticoids since we did not observe any effect on aldosterone production (data not shown). Altogether these data demonstrated that physiological concentrations of PGF_2α_ exerted an inhibitory effect on the endocrine functions of adrenal glands by inhibiting both catecholamine and corticosterone release.

## Discussion

PGF_2α_ has been shown to be an essential autocrine/paracrine regulator of ovarian steroidogenesis that initiates luteolysis and parturition in mammals (reviewed in [28]). Recently, PGF_2α_ was proposed to exert a repressive effect on testosterone release by testis Leydig cells [37], [38]. Although PGF_2α_ has this prominent role in the reproductive function, nothing was known about its possible impact on the adrenal gland. Furthermore, little was known about the biosynthetic enzymes involved in its selective production [29], [30]. In our previous work, we demonstrated that some aldo-keto reductase 1B (AKR1B) subfamily members expressed in the adrenal gland were endowed with high PGF synthase activity *in vitro* [26]. Our present work provides new insight in the understanding of PGF_2α_ biosynthesis and the role of this prostaglandin in the function of the adrenal gland. Indeed, we establish for the first time that PGF_2α_ acts as a negative regulator of adrenal endocrine functions and that the coordinate cell-specific regulation of both cyclooxygenases (COX1 and COX2) and aldo-keto reductases of the AKR1B subfamily (AKR1B7, AKR1B1, AKR1B3) could play a pivotal role in the generation of this signal.

Using *in vivo* approaches and murine cell culture models, we have demonstrated that both cortical (steroidogenic cells) and medullary (chromaffin cells) compartments of the adrenal gland secreted PGF_2α_ although the isoforms of COX and AKR1B involved in this biosynthetic pathway differed by their location and sensitivity to hormones. In good agreement with these differential expression patterns, PGF_2α_ secretion was found to be modulated by ACTH in steroidogenic cells and to be constitutive in chromaffin cells. Indeed, steroidogenic cells only expressed COX2 and two isoforms of the murine aldose reductases with patent PGF synthase activity *i.e.* AKR1B3 and AKR1B7. Among these enzymes, only COX2 and AKR1B7 were up-regulated by cAMP/ACTH. In particular, we found that ACTH induced a rapid (2h) and transient (persisting until 6–7 h and returning to near control level after 17 h) increase in COX2 protein. By contrast, chromaffin cells essentially expressed COX1 and AKR1B3 enzymes in a constitutive manner. Consistent with these observations, we have demonstrated that the cAMP/ACTH induction of PGF_2α_ release by steroidogenic cortical Y1 cells, was strictly dependent on the expression of AKR1B7 and resulted from a functional cellular coupling with COX2. These experiments provide evidence that AKR1B7 is a *bona fide* PGF synthase *in vivo*. In human, AKR1B1 was previously shown to be functionally related to the murine AKR1B7 [24]. Here, we showed that in the normal human adrenal gland, AKR1B1 and COX2 were co-localized in the steroidogenic cortical cells. Therefore, it seems likely that the human adrenal cortex could also have the potential to produce PGF_2α_ in response to ACTH. However, we observed an unexpected sustained expression of COX2 on sections of the human adrenal cortex. Although this might reflect constitutive COX2 expression in the human adrenal cortex, it seems more likely that this high level of COX2 expression reflects the high stress level of hospitalized patients. In this respect and in agreement with our results, stress induced by bacterial lipopolysaccharide injection in rat was shown to trigger a rapid COX2 mRNA expression in the adrenal cortex [39].

**Figure 6:**
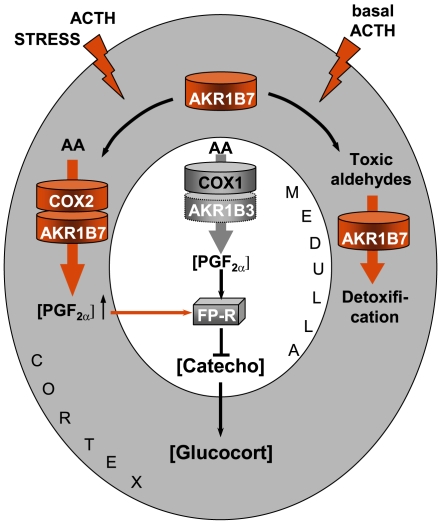
Proposed model to illustrate the integrated role of PGF_2α_ and PGFS of the AKR1B family in adrenal endocrine functions and cortico-medullary interactions. Free arachidonic acid (AA) is metabolized into PGH_2_ by COX enzymes and then converted into PGF_2α_ by PGFS of the AKR1B family. FP receptor expression is restricted to the medullary zone. PGF_2α_ synthesized in both the cortex and *medulla* thus signals in an autocrine/paracrine manner on chromaffin cells. This inhibits catecholamines production. Catecholamines produced in the *medulla* normally stimulate glucocorticoids release by the cortex. Decreased catecholamine production in response to PGF_2α_ stimulation thus results in a decrease in glucocorticoids production. The differential expression and regulation of both COX and AKR1B enzymes within the adrenal zones could allow the adjustment of PGF_2α_ production to limit stress response or control basal steroidogenesis by finely tuning glucocorticoid secretion. In basal conditions, chromaffin cells of the medullary zone constitutively secrete PGF_2α_, through the functional coupling between COX1 and possibly the PGFS AKR1B3. Under stress conditions, the resulting ACTH surge induces COX2 expression and sustains AKR1B7 levels in the cortex. Here, we demonstrated that the functional coupling between COX2 and AKR1B7 triggers a PGF_2α_ surge that could act as a local paracrine feedback to limit catecholamine-mediated glucocorticoid release. After the stress response has ended, COX2 returns to undetectable levels. The coupling between AKR1B7 and COX2 does not take place. AKR1B7 then functions only as a detoxifying enzyme of the harmful aldehydes produced under chronic/basal stimulation of steroidogenesis [23].

Although both steroidogenic and chromaffin cells of the murine adrenal gland secreted PGF_2α_, we showed that FP receptor expression was restricted to chromaffin cells. Consistent with this observation, high affinity binding sites for PGF_2α_ were only detected in the *medulla* within the bovine adrenal gland [40]. This suggested that both autocrine (within chromaffin cells) and paracrine (between steroidogenic and chromaffin cells) mechanisms were relaying PGF_2α_ effects. Our experimental observations confirm this hypothesis. First, PGF_2α_ had no effect on steroidogenesis when applied either to isolated murine adrenocortical cell lines or to rat primary cultures (data not shown) as could be expected for cells devoid of FP receptor. Second, by contrast, PGF_2α_ repressed both basal and glucocorticoid-induced catecholamine precursor release (dopamine) from a murine chromaffin cell line. Third, PGF_2α_ was shown to repress corticosterone release in a dose dependent manner when applied to isolated rat adrenals in perifusion experiments. Based on these experimental data, we propose that PGF_2α_ is involved in an intraadrenal regulatory loop that controls corticosterone release. The cortico-medullary interactions mediated by PGF_2α_ could be involved in both a paracrine feedback to limit stress response or in a local control of basal steroidogenesis. The proposed mechanisms are illustrated in Fig. 6 and would occur as follows: 1/A stress-induced ACTH surge triggers a transient induction of COX2 that couples with AKR1B7 in order to synthesize PGF_2α_ in the steroidogenic compartment. Increased amounts of PGF_2α_ inhibit glucocorticoid secretion through an indirect mechanism. This would involve at least repression of catecholamines release by chromaffin cells. These are known to promote the secretion of adrenocortical cells in a paracrine manner [41]. 2/In basal conditions, constitutive secretion of PGF_2α_ by chromaffin cells exerts an autocrine control on catecholamines release, thus limiting their paracrine action on adrenal steroidogenesis. By contrast, PGE_2_ was shown to promote the rapid release of glucocorticoid from isolated adrenal glands upon ACTH stimulation [42]. Hence, our results suggest that in the adrenal gland as in the female reproductive system [30], PGF_2α_/PGE_2_ exhibit opposite actions with corticostatic/corticotrophic and luteolytic/luteotrophic effects, respectively. But beyond this, our observations highlight an interesting paradox of PGF_2α_/PGE_2_ in fine tuning adrenal endocrine functions. Glucocorticoids are potent anti-inflammatory steroids. By inhibiting glucocorticoids production, adrenal PGF_2α_ limits anti-inflammatory signals, whereas PGE_2_, a classic pro-inflammatory mediator, increases anti-inflammatory signals by promoting their secretion [42]. Hence, the PGF_2α_/PGE_2_ paradigm could provide a plausible mechanism to the dichotomous action of PGs on the inflammatory process [43].

There are few examples of paracrine/autocrine factors that are involved in a negative control of glucocorticoid production by acting directly on the adrenal glands. These include the NT1-11 neurotensin fragment [44], urotensin-II (U-II) [45], leptin [46]–[48] and the Agouti-related protein (AgRP)[49], [50]. Among these peptides, NT1-11 is not produced by the adrenal and thus acts as an endocrine factor. By contrast, the three others may be synthesized within the gland. It is therefore possible that PGF_2α_-mediated activation of these repressive peptides could constitute an alternative (or a complementary) mechanism to PGF_2α_-mediated inhibitory tuning of glucocorticoid secretion.

By detoxifying aldehyde by-products of steroidogenesis [23] and by generating PGF_2α_ that inhibits adrenal steroidogenesis, AKR1B7 (and possibly AKR1B1 in human) has complementary actions aimed at protecting the endocrine activity of the adrenal cortex (Fig. 6). However, because of the two very different kinetic constants, PGF synthase or isocaproaldehyde reductase activities are likely to be recruited under different circumstances: the low affinity for isocaproaldehyde (*K*_m_ = 320 µM) combined to the highly cAMP/ACTH-dependent expression of AKR1B7 ensures an efficient reduction of isocaproaldehyde under chronic stimulation of steroidogenesis (high flow of substrate) with a limited risk of saturation [23]; on the other hand the high affinity for PGH_2_ (*K*_m_ = 4 µM) [26] combined with the acute cAMP/ACTH-dependent expression of COX2, allows a rapid response to stress. These two faces of a same enzyme are reminiscent of the 20α-hydroxysteroid dehydrogenase and PGF synthase activities of the bovine aldose reductase AKR1B5, that converge to initiate luteolysis in the bovine endometrium [25].

In summary, we have found that PGF_2α_ exerts a negative effect on the endocrine functions of both cortical and medullary zones of the adrenal gland. PGF_2α_-mediated inhibition of glucocorticoid production is indirect and could require the downregulation of catecholamine production. This would then result in downregulation of adrenal steroidogenesis. Alternatively, PGF_2α_ could up-regulate the expression of inhibitory peptides that remain to be identified. We provide evidence that this PGF_2α_ signal could originate from the differential expression and regulation of COX enzymes and aldo-keto reductases of the AKR1B family.

## Materials and Methods

### Experimental animals and hormonal manipulations

Animal studies were conducted in agreement with standards described by the NIH Guide for Care and Use of Laboratory Animals as well as with the local laws and regulations applicable to animal manipulations in France. For hormonal regulation studies, male mice of the B6D2 F1 strain (4-6-months old) were injected sc with vehicle (sesame oil), dexamethasone acetate for 5 days (75 µg twice daily; Sigma-Aldrich Corp., St. Louis, MO), or dexamethasone acetate (5 d) plus ACTH (10 µg/g, ip; Synacthene, Novartis Pharma S.A., Rueil-Malmaison, France) for the last 2 h or 17 h. For immunohistochemistry experiments, male mice were treated as described above, except for ACTH (1.2 U, im, daily, Synacthene, Novartis Pharma S.A., Rueil-Malmaison, France) for the last 6 h.

### Rat primary adrenal cells

Wistar male rats (150–200 g) were killed by decapitation. Adrenals were rapidly removed, and the *medulla* was separated from the cortex by squeezing the gland gently after removing the capsula. Cortical and medullary tissues were then separately incubated in HBSS for 20 min at 37°C in the presence of collagenase type I (Invitrogen, Cergy-Pontoise, France). Dispersed cells were pelleted and the remaining tissues were submitted to 3 or 4 repeated collagenase incubations until digestion was complete. Supernatants of viable cortical cells were pooled and seeded at a density of 500,000 cells/well in poly-D-lysine-coated 6-well plates (Sigma-Aldrich) and maintained at 37°C, 5% CO_2_ in Dulbecco's modified medium (DMEM)/Ham's F12, for 2 days. Medium was supplemented with 100 U/ml penicillin and 100 µg/ml streptomycin, 2.5% fetal calf serum (FCS), 2.5% horse serum (HS) and 1% insulin-transferrin-sodium selenite (ITS; Sigma-Aldrich). Dispersed chromaffin cells were pooled and seeded at a density of 300,000 cells in 6-well plates and maintained in DMEM for 2 days. Medium was supplemented with 100 U/ml penicillin and 100 µg/ml streptomycin and 10% FCS. On the third day, cells were incubated in serum-free medium for 24 hours. Cortical cells were treated with 10^−9^ M ACTH (Sigma-Aldrich) for 6 h or 12 h and culture media were removed for PGF_2α_ determination. Cortical cells and chromaffin cells were collected to perform western-blot experiments as described below.

### HEK293 cell lines expressing COX2 and AKR1B7 by stable transfection

HEK293 cells were transfected using a COX2 expression plasmid bearing neomycin resistance kindly provided by Dr M Murakami (Showas University, Tokyo, Japan) and clones were selected as previously described [32]. In order to establish cells transfected with both COX2 and AKR1B7, the clone expressing COX2 was transfected with the cDNA encoding AKR1B7 cloned in a pcDNA3.1/hygro vector (Invitrogen) or with the corresponding empty vector using Exgen (Euromedex, Souffelweyersheim, France). Briefly, 5 µg of plasmid were mixed with 20 µL of Exgen in 1 mL of 150 mM NaCl and added to cells at 70% confluence in 100 mm culture dishes. After selection in culture medium supplemented with hygromycin (40 µg/mL) for 4 weeks, single colonies were picked up and expanded. Expression of COX2 and AKR1B7 was assessed by western-blotting as described below.

### Cell line cultures

Y1 clones stably transfected with empty vector or AKR1B7 antisense cDNA [23] were maintained in DMEM/F12 supplemented with 10% FCS, 2 mM L-glutamine, 100 U/mL penicillin, 100 µg/mL streptomycin and 200 µg/mL G418 sulfate. For experiments, Y1 cells (1×10^6^) were seeded into 6-well plates. The next day, cells were placed in serum-free medium containing G418 sulfate in the absence or the presence of 10^−5^ M forskolin (Sigma-Aldrich) for 30 min to 6 h. MPC862L cells (kindly provided by Dr J. Powers, Tufts-New England Medical Center, Boston, MA) were maintained in RPMI 1640 supplemented with 10% HS, 5% FCS, 2 mM L-glutamine, 100 U/mL penicillin and 100 µg/mL streptomycin in dishes with collagen coating [51]. For experiments, MPC862L cells were treated in the same serum-free medium with or without 10^−6^ M dexamethasone (Sigma-Aldrich) or 10^−7^ M cloprostenol (Sigma-Aldrich) for 12 h. Stable transfected HEK293 cells were maintained in DMEM supplemented with 10% FCS, 2 mM L-glutamine, 100 U/mL penicillin, 100 µg/mL streptomycin, 200 µg/mL G418 sulfate and 20 µg/mL hygromycin. For cell activation, HEK293 cells (1×10^6^) were seeded into 6-well plates. After 2 days, the cells were placed in antibiotics-free medium supplemented with 1% FCS for 24 h. Cells were then stimulated for 30 min with 10 µM A23187 ionophore (Calbiochem, Darmstadt, Germany) in the same medium.

### Subcellular fractionation

Y1 cells (6×10^7^) were scraped gently in PBS-10% glycerol (v/v), washed in PBS and resuspended in 1 mL of hypotonic buffer (0.25 M sucrose, 20 mM Tris (pH 7.5), 10 mM KCl, 1.5 mM MgCl_2_) containing 1 X protease inhibitor cocktail (Complete, Roche Diagnostics, Meylan, France), 1 mM NaF and 1 mM VO_4_Na_3._ They were then incubated on ice for 30 min. After homogeneisation with a Dounce homogenizer (200–300 times), the lysate was subjected to centrifugation at 800 g for 5 min. The cytoplasmic supernatant was centrifuged at 10,000×g for 10 min at 4°C to obtain the heavy membrane pellet (HM). The remaining supernatant was centrifuged at 130,000×g for 1 hour at 4°C to obtain the light membrane pellet (LM) and the cytosolic supernatant. HM and LM fractions were resuspended in 2 volumes of Triton X-100 lysis buffer (150 mM NaCl, 10 mM Tris pH 7.5, 5 mM EDTA and 1% Triton X-100). Forty µg of total protein from each fraction were analyzed by western-blotting as described below.

### Western-blot experiments

Western-blots were performed as previously described [31]. Blots were incubated overnight at 4°C with the following antibodies: rabbit anti-COX1 (Cayman, Ann Arbor, MI, 1∶500), rabbit anti-COX2 (Cayman, 1∶1000), rabbit anti-FP receptor (Cayman, 1∶500), rabbit anti-StAR (1∶5000) [52], rabbit anti-AKR1B3 (L5, 1∶2000), rabbit anti-AKR1B7/B1 (L3, 1∶5000), rabbit anti-AKR1B8 (L7, 1∶2000), rabbit anti-Tyrosine Hydroxylase (TH) (Chemicon, Hampshire, UK, 1∶10,000), rabbit anti-SF1 (1∶2000) [53] or rabbit anti-β-actin (Sigma-Aldrich, 1∶3000). Primary antibodies were detected with a secondary antibody conjugated to peroxidase (PARIS, Compiegne, France, 1∶10,000). Peroxidase activity was detected with the enhanced chemoluminescent system (ECL, Perkin-Elmer,Courtaboeuf, France). For the production of antibodies directed against AKR1B isoforms, rabbits were injected with a glutathione S-transferase fusion of the 17 C-terminal amino acid residues of the murine AKR1B3, AKR1B7 and AKR1B8 proteins and the antibodies were obtained and tested as previously described [54]. L7 rabbit antiserum specificity, was increased by incubation on a GSTrap column (GE Healthcare, Orsay, France) harbouring the GST-AKR1B7 fusion protein. L3 antiserum is specific for both murine AKR1B7 and human AKR1B1, as previously described [24].

### Immunohistochemistry experiments

Human adrenal sections were kindly provided by the Cortico et MEdullosurrénale: Etude des Tumeurs Endocrines (COMETE) network. Adrenal tissue was obtained from normal glands removed during the surgery of adjacent non-endocrine tumours. Informed consent was given for adrenal tissue collection as part of a protocol approved by the Institutional Review Board of the Cochin Hospital. Mice were killed by vertebral dislocation and adrenal glands were immediately removed and fixed in Bouin's solution at 4°C overnight. After embedding in paraffin, tissues were sectioned (10 µm) and mounted on slides. Sections were then deparaffinized in toluene and rehydrated in ethanol with increasing concentrations of water. Quenching of endogenous peroxidase activity, incubation with antibodies and peroxidase staining were performed according to the manufacturer's instructions (Vectastain ABC kit and NovaRED substrate kit, Vector Laboratories, Burlingame, CA). Tissue sections were exposed to anti-COX2 antibody (Cayman, 1∶250), anti-AKR1B7/B1 antiserum (L3, 1∶500), anti-StAR antiserum (1∶500) and anti-TH antibody (Chemicon, 1∶500) at 4°C overnight. Tissues were counterstained with Harry's hematoxyline and mounted with Crystal Mount (Sigma-Aldrich).

### Quantification of adrenal PGF_2α_ content

Quantification was performed on pairs of adrenal glands from nine male mice (4–6 month-old). Individual adrenal weight was 3.5±0.6 mg. Deuterated standards (10 ng of d4-PGF_2α_ from Cayman) were added to homogenized adrenal tissue samples (2 glands) and acetic acid was added to obtain pH 3. Extraction was performed three times with 4 ml of ethyl acetate. After each extraction, the supernatants containing PGs were pooled and dried under nitrogen. The extracted sample was derivatized into PG-pentafluobenzylesters and separated by TLC with chloroforme/ethanol 93∶7 (v/v) as mobile phase. The silica areas containing PGs derivatives of interest was scrapped off and extracted three times with 2 mL of ethyl acetate/methanol 4∶1 (v/v). An other derivatization was performed to obtain pentafluorobenzylester-trimetylsilylether derivatives. Products of interest were then analyzed by gas chromatography–mass spectrometry (GC–MS Agilent Technologies). The quantification was done using selected ion monitoring according to the NICI mass spectral data obtained with standards. Ions at *m*/*z* 569 versus 573 were monitored for unlabeled and deuterated PGs, respectively.

### Analysis of PGF_2α_ release

PGF_2α_ release was measured in 6 h or 12 h culture media. Cellular debris were removed by centrifugation at 15,000×g and PGF_2α_ was quantified with a PGF_2α_ ELISA test kit (Neogen, Lexington, KY) according to the manufacturer's instructions.

### Analysis of catecholamine release

Dopamine release was measured in 12 h culture media of MPC862L chromaffin line by HPLC analysis with the Chromsystems kit (München, Germany).

### Perifusion experiments

The effects of PGF_2α_ (Sigma-Aldrich) and cloprostenol (Sigma-Aldrich) on corticosterone secretion were studied by a perifusion technique, as described previously [55]. Briefly, slices of rat adrenal cortex were layered between several beds of Bio-Gel P2 (Bio-Rad Laboratories, Inc., Richmond, CA) into perifusion chambers (equivalent of two adrenal glands/chamber). Adrenal tissue was continuously perifused with CO2-saturated HBSS at a constant flow rate (300 µl/min) and temperature (37°C). The glands were allowed to stabilize for 5 h to reach a steady-state level of corticosterone secretion, before any test substance was added. After stabilization, the mean secretion rate of corticosterone in basal conditions was 251±14 pg/min per adrenal. Test compounds were dissolved in CO2-saturated HBSS immediately before use and infused into the columns at the same flow rate as HBSS alone. This was achieved through a multichannel peristaltic pump (Desaga, Heidelberg, Germany). Effluent fractions were collected every 5 min (1.5 ml/fraction), and the tubes were immediately frozen until corticosterone assay. Corticosterone concentration was determined by RIA, without prior extraction, in 100 µl aliquots from each fraction. Sensitivity thresholds of the assays were 20 pg. The intra- and interassay coefficients of variation were 3 and 6%. Each perifusion pattern was established as the mean profile of corticosteroid production (±SEM) calculated from at least six independent experiments. Corticosterone levels were expressed as percentages of the basal values, calculated as the mean of eight samples (40 min), taken just before the infusion of test substances. The concentration-response curves were fitted using the Prism program (GraphPad Software, Inc., San Diego, CA).

### Statistical analysis

For PGF_2α_ and catecholamines quantification in culture media, statistical analyses were performed by a Student's *t* test. Values of *P*<0.05 and 0.01 were considered significant and highly significant, respectively. For corticosterone assay in organ perifusion experiments, statistical significance was assessed by Bonferroni test after one-way ANOVA. Value of *P*<0.001 was considered highly significant.
